# HDL biodistribution and brain receptors in zebrafish, using HDLs as vectors for targeting endothelial cells and neural progenitors

**DOI:** 10.1038/s41598-021-85183-9

**Published:** 2021-03-19

**Authors:** Nora Cassam Sulliman, Batoul Ghaddar, Laura Gence, Jessica Patche, Sepand Rastegar, Olivier Meilhac, Nicolas Diotel

**Affiliations:** 1 Université de La Réunion, INSERM, UMR 1188, Diabète Athérothrombose Thérapies Réunion Océan Indien (DéTROI), Saint-Denis de La Réunion, France; 2 Institute of Biological and Chemical Systems-Biological Information Processing (IBCS-BIP), Karlsruhe Institute of Technology (KIT), Postfach 3640, 76021 Karlsruhe, Germany; 3 CHU de La Réunion, Saint-Denis de La Réunion, France

**Keywords:** Neuroscience, Physiology, Stem cells

## Abstract

High density lipoproteins (HDLs) display pleiotropic functions such as anti-inflammatory, antioxidant, anti-protease, and anti-apoptotic properties. These effects are mediated by four main receptors: SCARB1 (SR-BI), ABCA1, ABCG1, and CD36. Recently, HDLs have emerged for their potential involvement in brain functions, considering their epidemiological links with cognition, depression, and brain plasticity. However, their role in the brain is not well understood. Given that the zebrafish is a well-recognized model for studying brain plasticity, metabolic disorders, and apolipoproteins, it could represent a good model for investigating the role of HDLs in brain homeostasis. By analyzing RNA sequencing data sets and performing in situ hybridization, we demonstrated the wide expression of *scarb1, abca1a, abca1b, abcg1,* and *cd36* in the brain of adult zebrafish. *Scarb1* gene expression was detected in neural stem cells (NSCs), suggesting a possible role of HDLs in NSC activity. Accordingly, intracerebroventricular injection of HDLs leads to their uptake by NSCs without modulating their proliferation. Next, we studied the biodistribution of HDLs in the zebrafish body. In homeostatic conditions, intraperitoneal injection of HDLs led to their accumulation in the liver, kidneys, and cerebral endothelial cells in zebrafish, similar to that observed in mice. After telencephalic injury, HDLs were diffused within the damaged parenchyma and were taken up by ventricular cells, including NSCs. However, they failed to modulate the recruitment of microglia cells at the injury site and the injury-induced proliferation of NSCs. In conclusion, our results clearly show a functional HDL uptake process involving several receptors that may impact brain homeostasis and suggest the use of HDLs as delivery vectors to target NSCs for drug delivery to boost their neurogenic activity.

## Introduction

High-density lipoproteins (HDLs) are complex particles composed of cholesterol (free and esterified), phospholipids, sphingolipids, triglycerides, and several proteins, including apolipoprotein A-I (ApoA-I), which is the most abundant^1,2^. HDLs constitute heterogeneous lipoprotein particles in terms of density (1.063–1.210 g/mL) and size (7–20 nm)^3,4^. Their main function is to ensure the clearance of cholesterol by promoting its reverse transport from the tissues back to the liver. However, they also exert pleiotropic functions, such as antioxidant, anti-inflammatory, anti-thrombotic, anti-proteolytic, and anti-microbial properties^2,5–8^. For decades, HDLs have been studied for their role in cholesterol metabolism and cardiovascular physiology, HDLs and/or ApoA-I levels being inversely correlated with the development of cardiovascular diseases, including atherosclerosis and stroke, in humans^9,10^.

HDLs exert their effects through four main receptors: scavenger receptor B type I (SCARB1 or SR-BI), ATP-binding cassette transporters ABCA1 and ABCG1, and the cluster of differentiation 36 (CD36)^11–13^. ABCA1 is a membrane protein interacting with lipid-free ApoA-I. It promotes cellular phospholipid and cholesterol efflux towards the extracellular free ApoA-I, leading to the formation of ApoA-I nanodiscs^14^. ABCG1 has high sequence homology with ABCA1 and interacts with mature HDL particles. It is the main transporter in charge of cholesterol and oxysterol efflux from human aortic endothelial cells, and it enhances endothelial nitric oxide synthase (eNOS) activity^14^. CD36, another receptor for HDLs, is a multiligand glycoprotein structurally related to SCARB1. It has a high affinity for native lipoproteins, including HDL, LDL, and VLDL, and for modified LDLs, such as oxidized and acetylated LDL^13^. SCARB1 (SR-BI) is a membrane glycoprotein that can recognize ApoA-I in HDL particles, as well as other apolipoproteins, that are free or in association with phospholipids. HDL particles preferentially bind to SR-BI, allowing selective uptake of cholesterol esters.

Interestingly, beyond the canonical metabolism and cardiovascular fields, plasma HDLs were more recently documented for their potential links with the central nervous system (CNS). Moreover, low HDL-cholesterol levels are correlated with neurodegenerative diseases, cognitive impairments, and depressive behaviors^3,15–18^. As well, many studies have pointed out the potential involvement of HDLs in neuroprotection after brain ischemia in mammals, in particular by exerting protective effects on the blood–brain barrier (BBB)^10^. In a rat thrombo-embolic model of stroke, intravenous injection of HDLs immediately or up to 3 h after the onset of stroke resulted in decreased infarct volume and reduced mortality^19^. Additionally, in both thrombo-embolic and monofilament models of focal middle cerebral artery occlusion (MCAO), HDL injection reduced hemorrhagic complications and improved the survival of rats treated with tissue plasminogen activator (tPA)^20^. Such neuroprotective effects were also observed in ischemic and excitotoxic lesions^21^. At the acute phase of stroke, HDL particles are also dysfunctional, displaying decreased anti-inflammatory and antioxidant activities, and are larger than in normal conditions^22^.

HDL receptors are widely expressed in the brain^23,24^. RNA sequencing analyses of the mouse cerebral cortex demonstrated the expression of all HDL receptors in neurons, microglial cells, astrocytes, oligodendrocytes, and endothelial cells^25^. Most HDL receptors appeared to be at least expressed in neurogenic regions, including the hippocampus^24^, and gene expression analysis in neural progenitor cultures demonstrated the enrichment of some HDL receptors in neural stem cells (NSCs), such as *Scarb1*^26^. Notably, the brain also appears to be an important site for the production of HDLs, with the most abundant apolipoproteins being ApoE instead of ApoA-I^27^. These data argue for the role of HDLs in brain plasticity and NSC activity and/or metabolism.

In recent years, zebrafish has become an emerging model for studying apolipoproteins, metabolism, and associated disorders, such as diabetes, obesity, and dyslipidemia, as well as brain plasticity^28–41^. Moreover, zebrafish express (1) all the main apolipoproteins sharing a high homology with human ones, (2) the major lipid transporters, and (3) the enzymes involved in lipoprotein metabolism, such as CETP, which is not expressed in rodents^35,42^. Cholesterol transporters Abca1 and Abcg1^42^, as well as Scarb1 (SR-BI) (www.ensembl.org) and Cd36^43^, have been found in zebrafish. In addition, in contrast to mammals that have only two main cerebral neurogenic niches during adulthood and a limited capacity to repair the brain, adult zebrafish exhibit numerous neurogenic niches across their brain and an important ability for healing^41,44–48^. Consequently, more and more studies investigate the impact of metabolic disorders, such as hyperglycemia and obesity, on brain homeostasis and plasticity^29,31,49,50^. However, there are only a few studies concerning HDL receptor expression in zebrafish, in particular in the central nervous system, and no data regarding their potential involvement in brain plasticity and neurogenesis.

Due to its high homology with the human genome, zebrafish is increasingly used as an interesting model for the study of human diseases and physiological processes^51^. Zebrafish also exhibit strong evolutionarily conserved similarities with humans concerning metabolic diseases, lipid and lipoprotein metabolism, and neurogenic properties^32,35,41,42,52,53^. These intrinsic features make the zebrafish an interesting candidate to study the impact of HDLs on the central nervous system in homeostatic and injury conditions before testing their neuroprotective effects on pre-clinical models of brain injury (i.e., brain ischemia in mice). The aim of this study was to investigate the potential role of HDLs on brain plasticity and brain repair mechanisms. For that, we first analyzed RNA sequencing data sets and cloned HDL receptor genes to determine their expression and distribution by in situ hybridization in the brain of adult zebrafish. Next, we investigated the role of HDLs in basal neurogenesis and brain repair mechanisms following a stab wound injury of the telencephalon. For that purpose, the biodistribution of HDLs and their capacity to reach the cerebral microvasculature, as well as the nervous tissue, in constitutive and regenerative conditions were investigated. This work supports the use of zebrafish for testing the neuroprotective capacity of HDL particles used as vectors after enrichment with drugs to favor brain plasticity and repair.

## Material and methods

### Ethics and animals

This study was conducted in accordance with the French and European Community guidelines for the use of animals in research (86/609/EEC and 2010/63/EU) and approved by the local Ethics Committee from the CYROI platform for animal experimentation (APAFIS#2018040507397248_v3; APAFIS# 20200908140689_v5).

For zebrafish (*Danio rerio*), adult (3–6 months-old) male and female wild-type (WT), tg(fli1:EGFP)^54^, and tg(GFAP::GFP)^55^ were maintained under standard conditions on a 14/10-h (h) light–dark cycle at 28.5 °C, and were fed daily with commercially available dry food (Gemma 300, Skretting). Fish were from the AB strain: WT, tg(GFAP::GFP), and tg(fli1:EGFP).

For mice (*Mus musculus*), C57BL/6J male (8 week-old, 25 g) mice were purchased from JANVIER LABS (Le Genest-Saint-Isle, France). They were maintained under standard conditions of light, temperature, and humidity and fed a standard diet ad libitum.

### Intraperitoneal injection of HDLs and HDL biodistribution in homeostatic conditions

For studying the biodistribution of HDLs in zebrafish (WT and fli:EGFP) and mice (WT) in homeostatic conditions, zebrafish and mice were intraperitoneally injected with 80 mg/kg of human plasma HDLs (plHDLs), reconstituted HDL solution (CSL BEHRING, rHDLs), or 1X PBS (vehicle) for controls.

For zebrafish, animals were anesthetized with 0.02% tricaine, weighed, and subjected to intraperitoneal injection of 1X PBS (vehicle) or HDLs for reaching a final concentration of 80 mg/kg of zebrafish bodyweight. The intraperitoneal injection of HDLs was performed using a 50 µl Hamilton syringe equipped with a sterile needle (BD Microlance 3; 30 G ½ ‘’; 0.3 × 13 mm). This syringe allows the precise injection of very low volumes in zebrafish as discussed in^56,57^. After anesthesia, fish were briefly weighed to determine the appropriate volume of the injection. For instance, 10 µl of HDLs at 1.6 µg/µl were injected in a 0.2 g fish to reach a final concentration of 80 mg/kg. According to the weight of fish (0.2–0.3 g), the volume injected varied from 10 to 12.5 µl. Fish were killed and fixed 1 h 30 min after injection.

For mice, intraperitoneal injections of HDLs (80 mg/kg of bodyweight) and PBS were performed, and the animals were allowed to survive 1 h 30 min post-injection.

The biodistribution study was performed in three independent experiments (n = 3 animals per condition: plHDLs, rHDLs, and vehicle) in both mice and fish.

### Stab wound of adult zebrafish telencephalon

Zebrafish were anesthetized with 0.02% tricaine and subjected to mechanical injury of the telencephalon. Mechanical injury to the telencephalon was performed by inserting a sterile needle (BD Microlance 3; 30 G ½ ‘’; 0.3 × 13 mm) in the right telencephalic hemisphere, following a dorsoventral axis and guided by landmarks on the head, as previously described^40,58^. After this procedure, fish were put back into their respective tanks and allowed to survive for different kinetic times before being processed for gene expression analyses and/or histological analyses.

### Biodistribution of HDLs in a brain injury context

HDL biodistribution in brain injury conditions was evaluated by intraperitoneal injection (80 mg/kg) of rHDLs either 30 min before the stab wound injury (n = 4 for rHDLs; n = 3 for PBS) or immediately after the lesion (n = 4 for rHDLs; n = 3 for PBS). In both conditions, fish were sacrificed 1 h 30 min after the injection. The results presented are from two independent experiments.

To test the effects of HDLs on microglial recruitment and injury-induced proliferation, an intraperitoneal injection (80 mg/kg) of plHDLs was performed immediately after stab wound injury of the telencephalon. Fish were then allowed to survive for 2- and 5-days post lesion (dpl) before analysis of microglia activation and ventricular proliferation, respectively. For each time point, a total number of nine fish were injected with plHDLs and ten fish with PBS; these numbers corresponded to two independent experiments.

### Intracerebroventricular injection of HDLs

To determine the effects of HDLs on ventricular cell proliferation, intracerebroventricular injections of plHDLs or rHDLS were performed. For this purpose, fish were anesthetized with 0.02% tricaine. A hole was made in the skull at the junction between the tel- and diencephalon as previously described^58^. Around 2 nanoliters of plHDLs (10 mg/ml) and rHDLs (20 mg/ml) were injected into the ventricle with a glass capillary using a microinjector (Femtojet, EPPENDORF). Another group of fish was also injected with the vehicle (PBS). Fish were then allowed to recover before being killed 24 h post-injection and fixed for proliferative analysis. The total number of injected fish from two independent experiments were: n = 6 fish for PBS, n = 6 for plHDLs, and n = 4 for rHDLs.

For testing the capacity of HDL to deliver molecules of interest, a fluorescent dye (DilC18) was incorporated into HDLs. Intracerebroventricular injections of HDLs were performed (n = 3) and fish were sacrificed 1 h 30 min post-injection and processed for confocal microscopy.

### Tissue sampling and processing

For zebrafish, animals were euthanatized using 0.02% tricaine before being decapitated. Brains and/or telencephalon were carefully removed and frozen for gene expression analysis. Alternatively, fish were fixed overnight at 4 °C in 4% paraformaldehyde (PFA) in PBS (pH 7.4) before dissection of brain, liver, and kidney tissues for immunohistochemistry (IHC) and/or in situ hybridization (ISH) analyses according to previous studies^58–60^.

For mice, animals were anesthetized with Isoflurane (ISOFLO Centravet France) and killed by intracardiac puncture before intracardiac perfusion with PBS and fixation with 4% PFA. Brain, liver, and kidney tissues were subsequently processed for cryostat embedding and cutting.

### Brain RNA extraction, reverse transcription, qPCR, and RNA sequencing analyses

Total brain and/or telencephalon RNA were extracted with the TRIzol reagent after tissue homogenization with the QIAGEN Tissue Lyser II (90 s at 250 rpm). Two micrograms of RNA were reverse transcribed to cDNA using random hexamer primers (Life Technologies) and MMLV reverse transcriptase (INVITROGEN, REF: 28025-021) following the manufacturer’s instructions.

For qPCR analysis, experiments on three pools of five telencephala were performed on an AB7500 real-time PCR system (Applied Biosystems, Foster City, CA) with SYBR green master mix (Eurogentec). Specific zebrafish primers were used (Table 1). The relative expression of the *HDL receptor* genes was normalized against the expression level of the *ef1a* gene.

**Table 1:** qPCR primers.

Zebrafish	Forward	Reverse	Amplicon size
*abca1a*	AAGGAACAATCATTAAGGAGCAA	CCACAACAGTAAACCCAACTGA	108
*abca1b*	GCCATGGTACTTCCCTTTCA	TCCTCTTCAATGCAAACAGC	111
*cd36*	TGGTCTTCTTGACATTACTTCCTG	CATCTAAGTTGGGGTTCATTCC	119
*scarb1*	GTCAGCTCCTGCAGACACG	TCTTCACTTGGGCTCAATCC	107
*abcg1*	TCTTCAGAGGGATGCCACA	CCGCAAGTGAGTCAACACC	111
*ef1α*	AGCAGCAGCTGAGGAGTGAT	CCGCATTTGTAGATCAGATGG	140

For RNA sequencing analyses, data were reanalyzed from the work of Wong and Godwin for the whole brain (n = 4), and from the study of Rodriguez-Viales et al. and Gourain et al. for control telencephala and 5 dpl telencephala (n = 3)^58,61,62^.

### Cloning, probe synthesis, and in situ hybridization (ISH)

Standard PCR was performed using zebrafish brain cDNA and gene-specific primers to obtain PCR products of almost 500 bp that were subcloned into the pGEMT-Easy vector (PROMEGA, Madison, WI, USA) (Table 2). Plasmids were sequenced and linearized for the synthesis of digoxigenin (DIG) labeled antisense riboprobes using T7 or SP6 RNA polymerases.

**Table 2:** Cloning primers.

ZF gene	Forward	Reverse	Amplicon size
*abca1a*	TGAAGATGGGGAATCTCCTG	GCTCTTCCTCAATGCACACA	567
*abca1b*	TCCACCCAACGTCAACTACA	TGCTGATCAGGAAACACTGC	569
*abcg1*	ACGCAGTTCTGCATCCTCTT	ACAGGACCCACAAAAGTTGC	506
*cd36*	TACTTGCTGCCTGTTGATGC	ATTCGTTTTTGACGGTTTCG	543
*scarb1*	GGGGCTGTTCACCATCTTCA	ACCGAACAGAGACGTTCACC	515

Chromogenic ISH on whole adult brains was performed as described^48,59^. Briefly, brains were rehydrated and washed with PTW (0.1% Tween 20, PBS buffer; pH 7.4). Tissue permeabilization was performed using proteinase K (10 μg/ml) diluted in PTW (30 min at 20 °C). Brains were subsequently post-fixed in 4% PFA and carefully washed. After a prehybridization step (3 h at 65 °C), the brains were incubated overnight with the DIG-labeled probes in hybridization buffer (pH 6) at 65 °C. The next day, they were washed, and 50 μm-thick sections were cut using a vibratome (LEICA VT1000S). Brain sections were incubated overnight at 4 °C with anti-digoxigenin-AP, Fab fragments (1:2000; ROCHE; Cat# 11093274910). Finally, the sections were stained with NBT/BCIP substrate (pH 9.5). At least five brains were investigated for each transcript studied: *scarb1* (n = 5), *abca1a* (n = 7), *abca1b* (n = 8), *abcg1* (n = 5), and *cd36* (n = 7). Three independent experiments were performed.

For *scarb1* fluorescence ISH, tyramide amplification was performed (TSA Plus Cyanine 3 System, PERKIN ELMER, Boston, MA) using DIG-POD (poly) antibody (1:1,000, ROCHE) on three brains as described in^58,60^.

### Immunohistochemistry

For zebrafish brain immunostaining, vibratome sections were made. Briefly, fixed tissues were dehydrated in 100% MetOH and kept at − 20 °C at least overnight. Then, samples were rehydrated, permeabilized with PTW (1X PBS containing 0.1% Tween 20), and 50 μm-thick sections were cut using a vibratome (LEICA VT 1000S). After blocking the sections for 2 h in blocking buffer (PTW containing 0.2% BSA, 1% DMSO), incubation with primary antibodies was performed overnight at 4 °C. The next day, the brain sections were washed three times with PTW before being incubated with secondary antibodies and DAPI (1 ng/ml, THERMOFISCHER) for 2 h at room temperature. Finally, sections were washed in PTW and mounted on slides with Aqua-Poly/Mount (POLYSCIENCES).

For cryostat brain, liver, and kidney sections, mice and zebrafish tissues were cryoprotected in PBS containing 30% sucrose for one night at 4 °C. The next day, the tissues were embedded in Tissue-Tek OCT, frozen at − 80 °C and cut with a Leica CM 1520 cryotome at a 12 μm thickness. The slides were dried and then rehydrated twice in 1X PBS, permeabilized with PTW, and blocked for 45 min in blocking buffer. Incubation with primary antibodies was performed overnight at room temperature. The next day, slides were rinsed three times with PTW, and incubations with secondary antibodies and DAPI were performed for 1 h 30 min. Slides were washed three times in PBT and mounted with antifading medium Vectashield (H-1000, VECTOR LABORATORIES, Burlingame, CA).

The references and concentrations of the primary and secondary antibodies, as well as the DAPI nuclear counterstaining, used in this work are shown in Table 3.

**Table 3:** Antibodies for immunochemistry.

Primary antibody			
Antibodies	Dilution	Reference	Host
ApoA-I	1:100	CALBIOCHEM 178422	Rabbit
PCNA	1:100	DAKO M0879	Mouse
l-Plastin (Lcp1)	1:8000	Gift from M. Redd^63^	Rabbit
Aromatase B	1:600	Gift from F. Brion^37,64^	Rabbit
HuC/D	1:300	INVITROGEN, A21271, Clone 16A11	Mouse
Control Ig	As for ApoA-I	DAKO, X0903	Rabbit

Secondary antibody			
Antibodies or dye	Dilution	Reference	
Goat anti-rabbit Alexa 488	1:200	THERMOFISCHER A11008	
Goat anti-rabbit Alexa 594	1:200	THERMOFISCHER A11012	
Goat anti-mouse Alexa 488	1:200	THERMOFISCHER A11001	
Goat anti-mouse Alexa 594	1:200	THERMOFISCHER A11005	
DAPI (4′,6-diamidino-2-phenylindole, dihydrochloride)	1 ng/ml	THERMOFISCHER D1306	

Each IHC experiment was repeated in at least three independent experiments. For ApoA-I immunohistochemistry, a negative control was performed using IgG from rabbit (Negative CTRL Rabbit IgG, X0903, DAKO), diluted at the same concentration as the anti-ApoA-I antibodies. No staining was observed in these conditions in both fish and mice, reinforcing the specificity of ApoA-1 staining.

### Preparation of HDLs and HDL fluorescence labeling

Lipoproteins were isolated from a pool of EDTA-treated plasma of healthy male and female volunteers by ultracentrifugation. Briefly, the plasma density was adjusted to d = 1.22 with KBr (0.003 g/l) and overlaid with KBr saline solution (d = 1.063). Ultracentrifugation was performed at 100,000 × *g* for 20 h at 10 °C. The density of the bottom fraction containing HDLs was adjusted to 1.25 with KBr and overlaid with KBr saline solution (d = 1.22). The HDL fraction (top layer) was recovered as a single band and was desalted and concentrated by three washes with saline using a centrifugal concentrating device (cutoff 10 kDa; Vivascience, Stonehouse, UK). The protein concentration was determined by the BCA method (BCA Protein Assay Kit, Thermo Scientific Pierce).

For 1,1′-dioctadecyl-3,3,3′,3′-tetramethylindocarbocyanine perchlorate (DiI) labeling (REF: D3911; THERMOFISCHER), which labels the phospholipid moiety, 300 μg of DiI was added to 2 mg HDL in PBS overnight at 37 °C. Labeled HDL preparations were then re-isolated by ultracentrifugation to eliminate free DiI, and then it was sterile-filtered and stored at 4 °C until further use.

### Microscopy

Observations were carried out using a NanoZoomer S60 (HAMAMATSU) slide scanner, a Nikon eclipse 80i equipped with a Hamamatsu camera, and a confocal microscope (Confocal NIKON C2si) and its NIS elements software.

### Cell count

For analyzing brain cell proliferation following intracerebroventricular injection of HDLs, brains were fixed and processed for PCNA immunostaining. The PCNA positive area was counted in blind conditions on three vibratome sections/fish for each condition (PBS; rHDLs, plHDLs). Similarly, for analyzing the number of microglia and proliferative cells in the ventricular layer at 2 dpl and 5 dpl, respectively, three vibratome sections per fish were selected in the control and lesioned telencephalon.

Images were analyzed using the ImageJ software considering the area stained (along the ventricular layer for PCNA and in the brain parenchyma for l-plastin), and after adjusting the appropriate parameters (threshold, binary, and watershed). These analyses were done in blind conditions.

### Statistical analyses

Comparisons between groups were performed using a statistical Student t test or using one-way ANOVA for multiple comparisons using PRISM v7 from GRAPHPAD software company.

### ARRIVE guidelines

The study was carried out in compliance with ARRIVE guidelines.

## Results

### Expression of HDL receptors during zebrafish development

Given the few numbers of studies documenting HDL receptors in zebrafish, their expression was first investigated during zebrafish development looking at the mammalian homologues of *scarb1, abca1, abcg1,* and *cd36* (Suppl. Fig. 1). Reanalyzing a recently published RNA sequencing data set from^65^ that provided global transcriptomic profiling from the zygote stage (1-cell) to 5 days post-fertilization (dpf), we investigated the temporal gene expression of HDL receptors during embryogenesis. Briefly, *scarb1* was expressed from the zygote stage to larval day 5 with expression peaking at the blastula stage (128–1000 cells). In contrast, *abca1a* and *abca1b* were only barely detected until the blastula dome/gastrula 50%-epiboly stages. Expression of the *abca1a* gene reached a peak at 5 dpf, and *abca1b* gene expression was high until the pharyngula prim 15 stage. *abcg1* and *cd36* gene expressions were very low or undetectable until the larval protruding mouth stage, then it increased strongly to 5 dpf. These data also showed that *scarb1* displayed an important maternal contribution and that HDL receptors are dynamically expressed during zebrafish embryogenesis.

### HDL receptors are widely expressed in the brain of adult zebrafish

**Figure 1:**
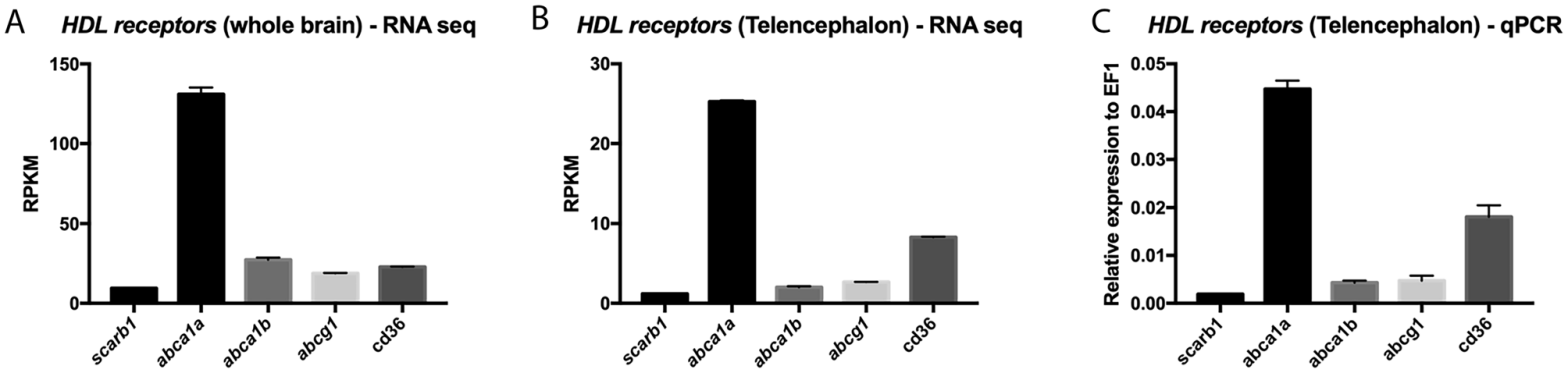
*HDL receptor* mRNA expression in the whole brain and in the telencephalon of adult zebrafish. RNA sequencing data showing the relative levels of expression of HDL receptors in the whole zebrafish brain (n = 4) (**A**) and in the zebrafish telencephalon (n = 3) (**B**) of adult animals (data from Rodriguez-Vialez et al. 2015; Gourain et al. 2020; Wong and Godwin 2015)^58,61,62^. (**C**) qPCR analysis from 3 pools of 5 telencephala, providing the relative gene expression of HDL receptors in the telencephalon. *RPKM* reads per kilobase million.

Despite the presence of the BBB, it was shown and/or suggested that some of the smaller circulating HDLs can enter the brains of humans and mice^66–68^. These data suggest a possible role for peripheral HDLs in the central nervous system. However, the cerebral expression and distribution of HDL receptors are not known in zebrafish. We consequently reanalyzed two different RNA sequencing data sets from the whole brain (Fig. 1A) and from telencephala (Fig. 1B) published by Rodriguez Viales et al., and Wong and Godwin, respectively. *HDL receptor* genes appeared to be significantly expressed in the brains of adult zebrafish^58,62^. Our own qPCR experiments also demonstrated the expression of these receptors in the telencephalon with similar relative expression of *HDL receptors* as the ones documented by RNA sequencing (Fig. 1C). *abca1* was the most highly expressed HDL receptor in the brain and telencephalon (Fig. 1). Considering the significant expression of HDL receptors in the brain of adult zebrafish, their distribution was further investigated by in situ hybridization (ISH).

**Figure 2:**
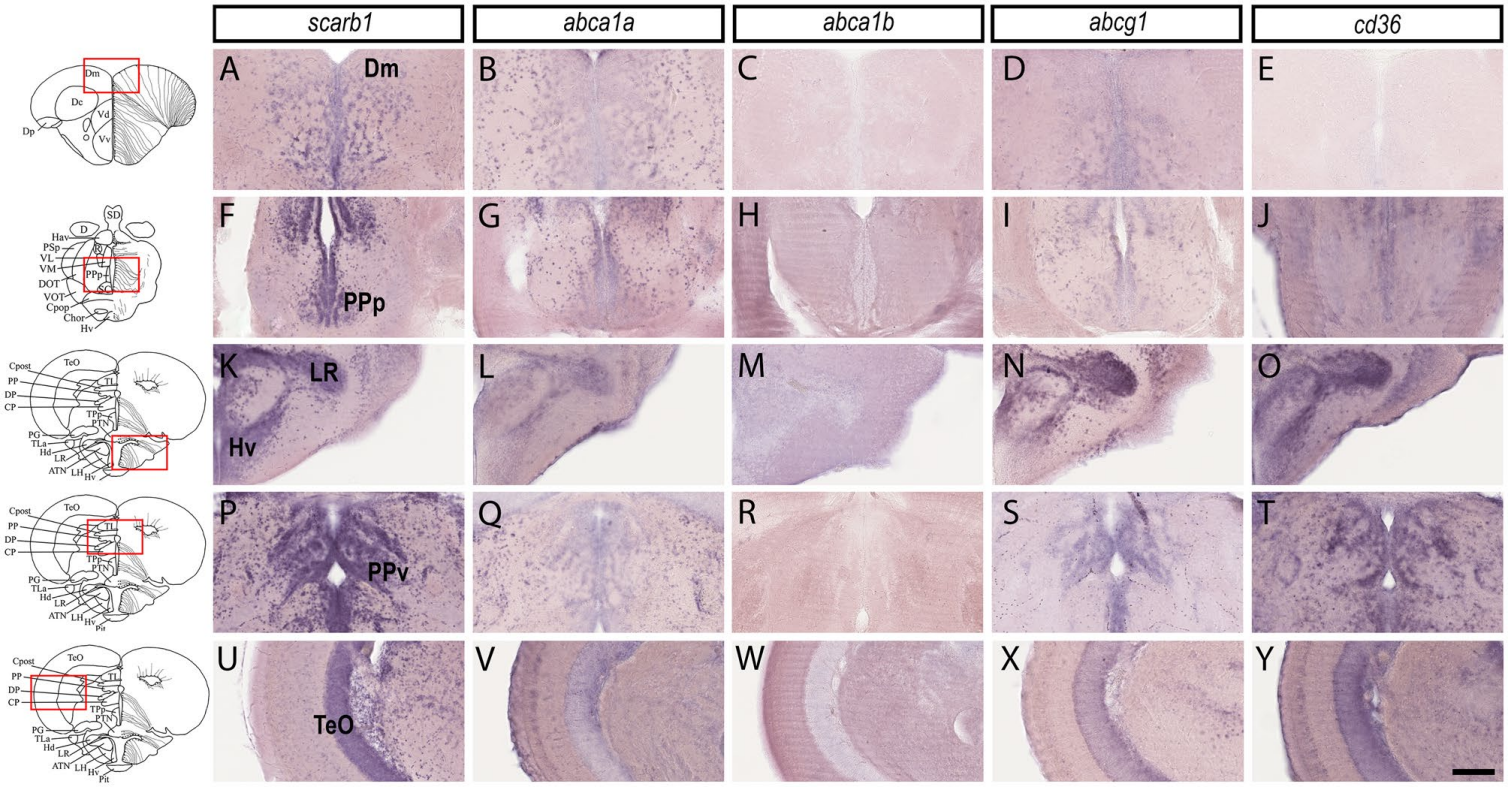
*HDL receptors* are expressed in the brain parenchyma and along the periventricular zones. In situ hybridization of *HDL receptors* in the brain of adult zebrafish. The scheme provides the localization of the transversal section performed and the red square shows the regions where the high-magnification views were made. In situ hybridization in the medial zone of telencephalic area (Dm, **A**–**E**), the posterior part of the preoptic area (PPp, **F**–**J**), the ventral zone of the periventricular hypothalamus (Hv, **K**–**O**), around the lateral recess of the diencephalic ventricle (Lr, **K**–**O**), the periventricular pretectal nucleus ventral part (PPv, **P**–**T**), and the optic tectum (TeO, **U**–**Y**). Note that most HDL receptors are expressed in the brain parenchyma where neurons are localized and in periventricular zones where NSCs are localized. Scale bar = 50 μm.

As a result, independent in situ hybridization experiments (n = 3–6) demonstrated a wide expression of *scarb1, abca1a, abcg1,* and *cd36* in the brain, while *abca1b* was almost not detected (Fig. 2). *scarb1*, *abca1a, cd36,* and *abcg1* were widely expressed in the whole brain from the junction between the telencephalon/olfactory bulbs to the cerebellum. In contrast, *abca1b* gene expression failed to be detected by in situ hybridization except in the cerebellum (data not shown). In summary, *scarb1*, *abca1a,* and *abcg1* displayed almost similar patterns and were detected in the dorsal (Vd), ventral (Vv), and central (Vc) nuclei of the ventral telencephalic area, as well as in the central zone (Dc) and medial zone (Dm) of the dorsal telencephalic area. In more posterior regions, these genes were expressed in the supra-(Vs) and post-commissural nucleus (Vp). In the diencephalon, all transcripts coding for HDL receptors (with the exception of *abca1b*) were detected in the anterior (PPa) and posterior (PPp) part of the preoptic and in the anterior (A), dorsal (DT), ventromedial (VM), and ventrolateral nuclei (VL) of the thalamus. In the rhombencephalon, *scarb1*, *abca1a, cd36,* and *abcg1* were reported in the posterior tuberal nucleus (PTN), the torus lateralis (TLa), and in the different hypothalamic nuclei (Hv, LH, LR, PR, Hc, Hd). They were also expressed in the nucleus of the inferior lobe (IL), optic tectum (TeO), torus longitudinal (TL), torus semi-circularis (TS), and valvula of the cerebellum and the cerebellum (VCe + Ce). The specificity of the staining was assessed by hybridization without a probe leading, to the absence of staining (data not shown), and with the use of a specific *id1* antisense probe as a positive control, resulting in a clear and obvious labeling along the ventricular layer as previously described (data not shown)^58,59^.

### *Scarb1* is expressed in neurons and radial glial cells behaving as neural stem cells

**Figure 3:**
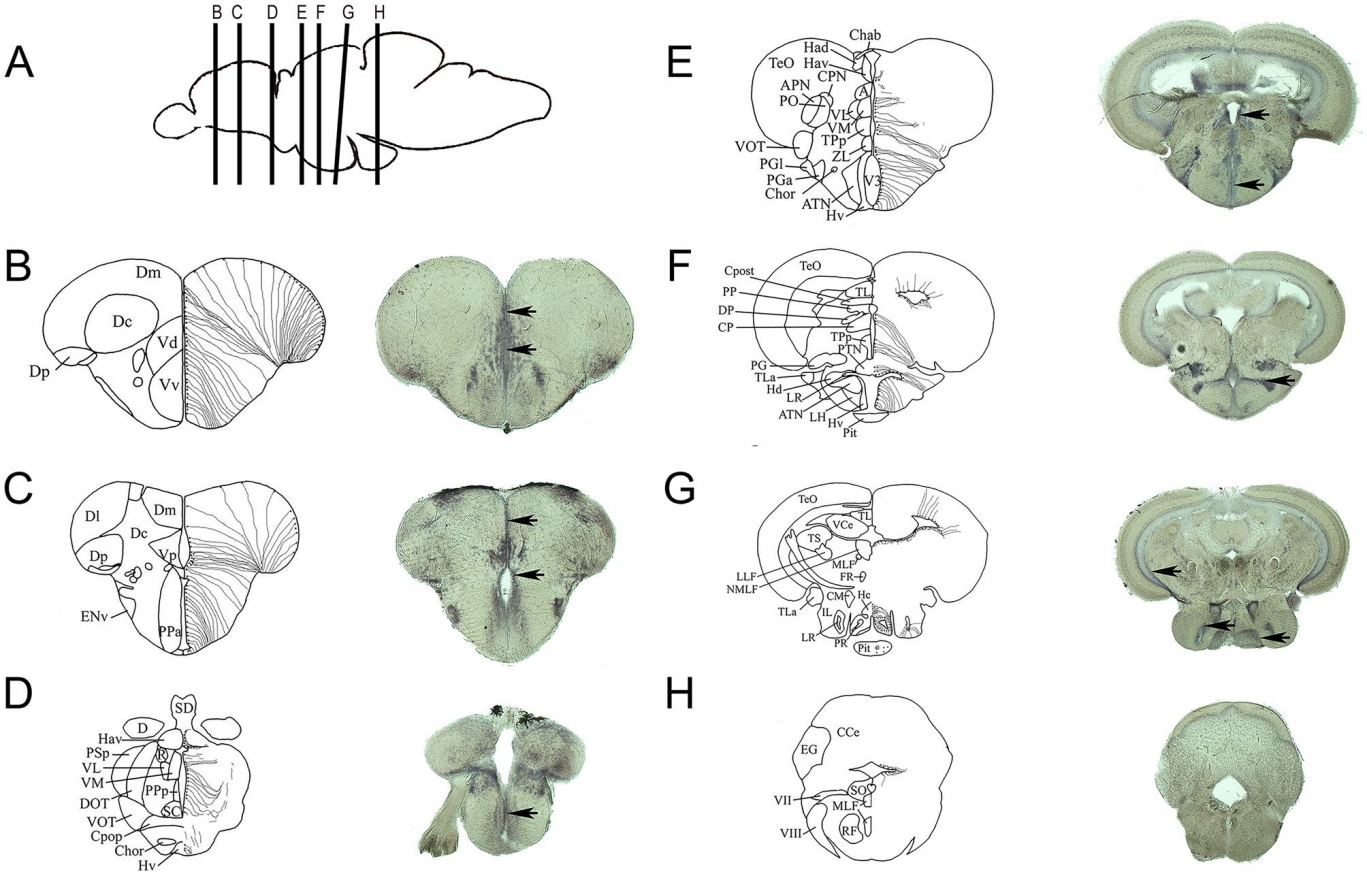
Relationship between *scarb1* expression and the distribution of radial glial cells. (**A**) Sagittal zebrafish brain view showing the respective transverse sections provided from B to H. (**B**–**H**) The schemes adapted from the zebrafish brain atlas and from Menuet et al.^70,71^ illustrate the transverse brain section and the different brain regions/nuclei (left part), as well as the localization of radial glial cells (neural stem cells) along the brain ventricles (right part). In situ hybridization at the level of the telencephalon (**B**, **C**), the anterior and posterior preoptic area (**C**, **D**), the anterior, medial, and caudal hypothalamus (**E**–**G**), as well as the medulla oblongata (**H**) demonstrate a wide *scarb1* expression in the brain parenchyma and along the ventricular layer where radial glia reside (Black arrows). Scale bar = 50 μm.

**Figure 4:**
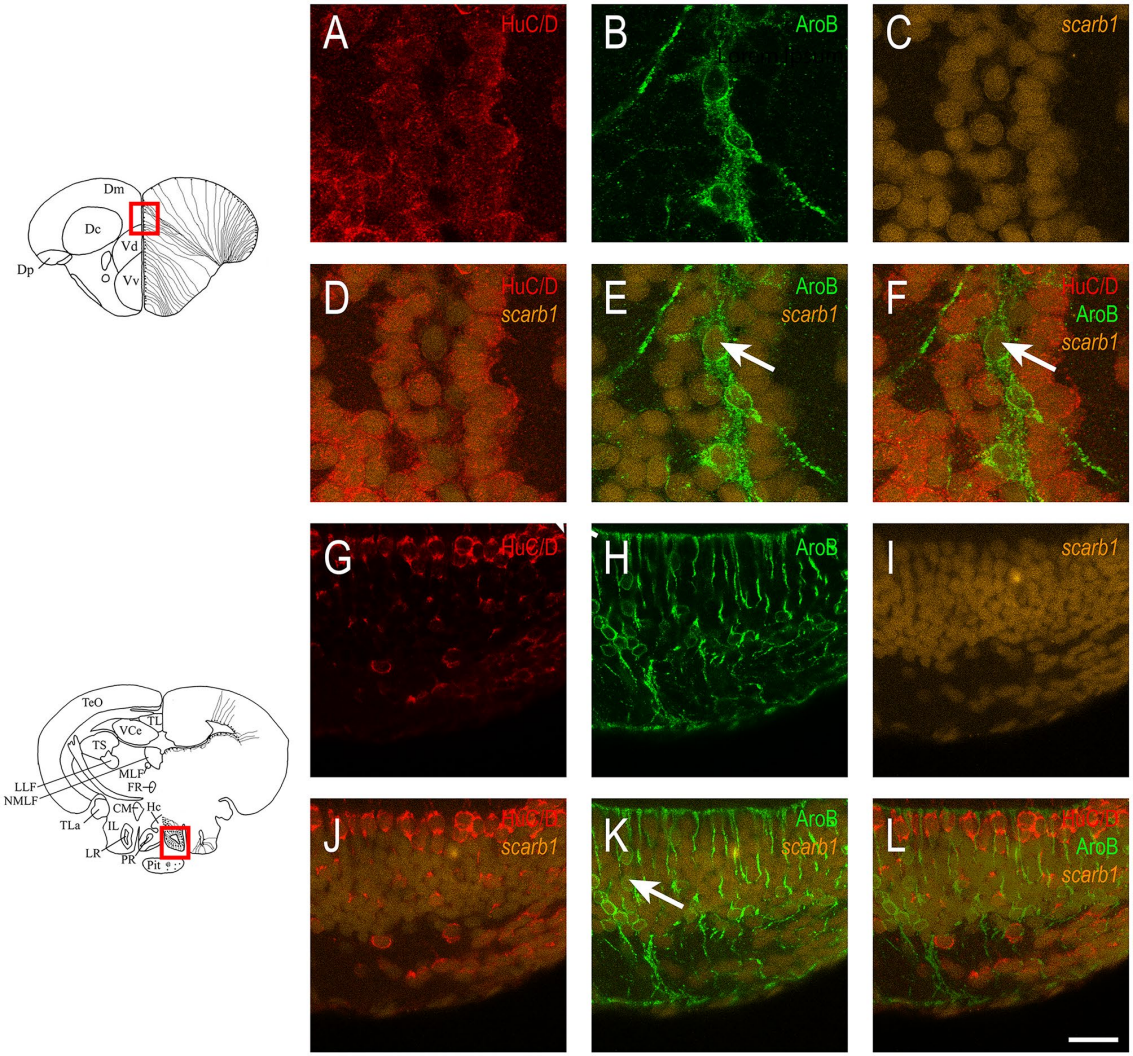
*Scarb1* is expressed in neurons and radial glial cells. Fluorescent *scarb1* in situ hybridization in the medial zone of telencephalic area (**A**–**F**) and in the caudal hypothalamus around the posterior recess of the diencephalic ventricle (**G**–**L**)**.** *Scarb1* in situ hybridization (orange) is followed by Aromatase B (green) and HuC/D (red) immunohistochemistry to label NSCs and neurons, respectively. Note that *scarb1* is expressed in both cell types. Scale bar = 50 μm.

Although the expression patterns of *scarb1*, *abca1a*, *abcg1,* and *cd36* displayed a wide and overlapping distribution, some differences were noticed. In contrast to the other receptors for which expression in the ventricular zone was very weak, *scarb1* expression was markedly detected in the ventricular zone, where radial glial cells behaving as NSCs are localized (Figs. 2 and 3, black arrows). Indeed, *scarb1*-positive cells were observed along the ventricles of the Vv, Vd, and Dm (Fig. 3B,C), the posterior part of the preoptic area (Fig. 3D), and the anterior (Fig. 3E), mediobasal (Fig. 3F), and caudal (Fig. 3G) hypothalamus. In addition, some *scarb1*-positive cells from the periglomerular gray zone and lining the ventricles of the optic tectum were observed (Fig. 3E–G). Taken together, such data strongly argue for *scarb1* expression in NSCs. To ascertain this hypothesis, fluorescent *scarb1* ISH was performed, followed by Aromatase B and HuC/D immunohistochemistry, allowing us to label NSCs (radial glia) and neurons, respectively^37,69^. As shown in the dorsomedial telencephalon and in the regions surrounding the posterior recess of the hypothalamus, *scarb1*-positive cells corresponded to both HuC/D-positive neurons and AroB-positive neural stem cells (Fig. 4). These results were also confirmed by performing fluorescence ISH using the anti-digoxigenin antibody conjugated with horseradish peroxidase (data not shown). Taken together, these observations strongly argue for a possible role of *scarb1* and HDL signaling in the control of NSC activity.

### Peripheral HDLs reach the brain microvasculature and the damaged brain tissue

To investigate the capacity of HDLs to target the brain, a biodistribution analysis was performed following intraperitoneal injection in zebrafish and was compared with mice. After 1 h 30 min, the two main metabolic organs, the liver and kidneys, were investigated for human ApoA-I detection by immunohistochemistry. Positive labeling was observed in the liver and kidneys of zebrafish and mice injected with both plasma HDLs and reconstituted HDL particles (Suppl. Fig. 2, green), while no labeling was observed with the negative control IgG or in PBS-injected animals.

**Figure 5:**
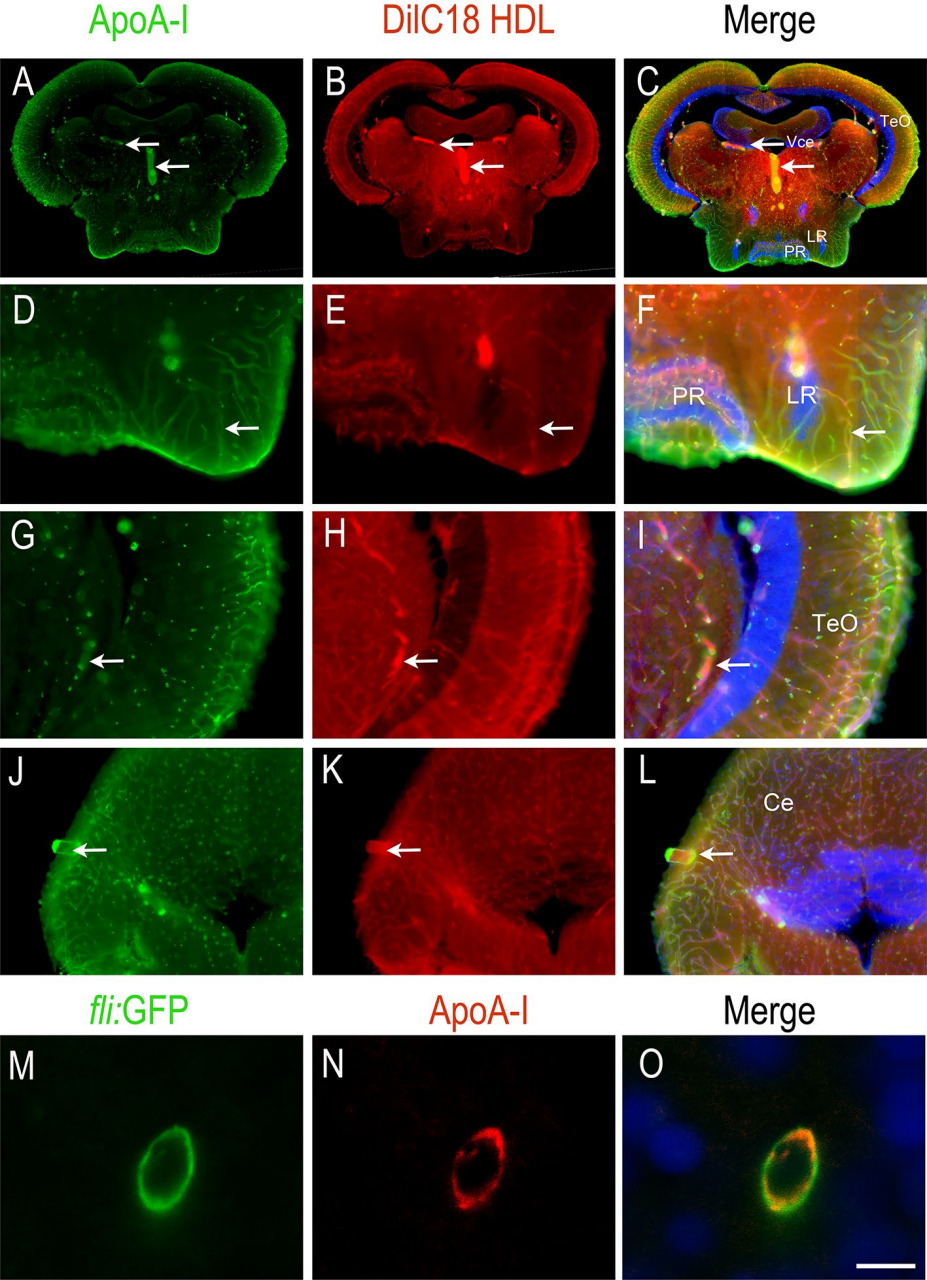
HDLs reach the brain microvasculature in zebrafish. ApoA-I immunohistochemistry (green) on zebrafish brain Sects. 1 h 30 min after intraperitoneal injection with 80 mg/kg of fluorescent HDL particles (red). Note that similar data were obtained with HDLs isolated from plasma (plHDL, n = 3) and with reconstituted HDLs (rHDL, n = 7). (**A**–**L**) ApoA-I staining (green) colocalizes with fluorescent HDLs (red) as clearly evidenced by the merged pictures and revealed blood vessel staining. (**M**–**O**) Confocal imaging of a transverse brain section from a Tg(fli1:EGFP) zebrafish expressing GFP in the endothelial cells from blood vessels^54^. Note ApoA-I staining (red) detection in endothelial cells (green). The merge pictures (**C**, **F**, **I**, **L**, **O**) also show cell nuclei using DAPI counterstaining (blue). Scale bar = 50 μm.

In the brain, HDLs were also documented to reach the cerebral blood flow and to be taken up by the cerebral endothelial cells during a stroke^19^. Using DilC18 HDLs, red fluorescence was observed in zebrafish and mice brain microvasculature 1 h 30 min after injection (Fig. 5). These results were confirmed by ApoA-I immunohistochemistry, showing overlapping staining with the DilC18 dye (Fig. 5). No signal was observed by incubation with control IgG or with ApoA-I immunochemistry in PBS-injected animals, demonstrating the specificity of the signal (Suppl. Fig. 3). Taking advantage of a *fli:*GFP transgenic fish, in which GFP is expressed in endothelial cells, we further demonstrated by performing ApoA-I immunohistochemistry that HDLs are taken up by endothelial cells from the brain vasculature (Fig. 5M–O). We did not observe any significant staining in the brain parenchyma under homeostatic conditions. Such labeling of the blood vessels was also observed in mice injected with plHDLs and rHDLs (Suppl. Fig. 4).

**Figure 6:**
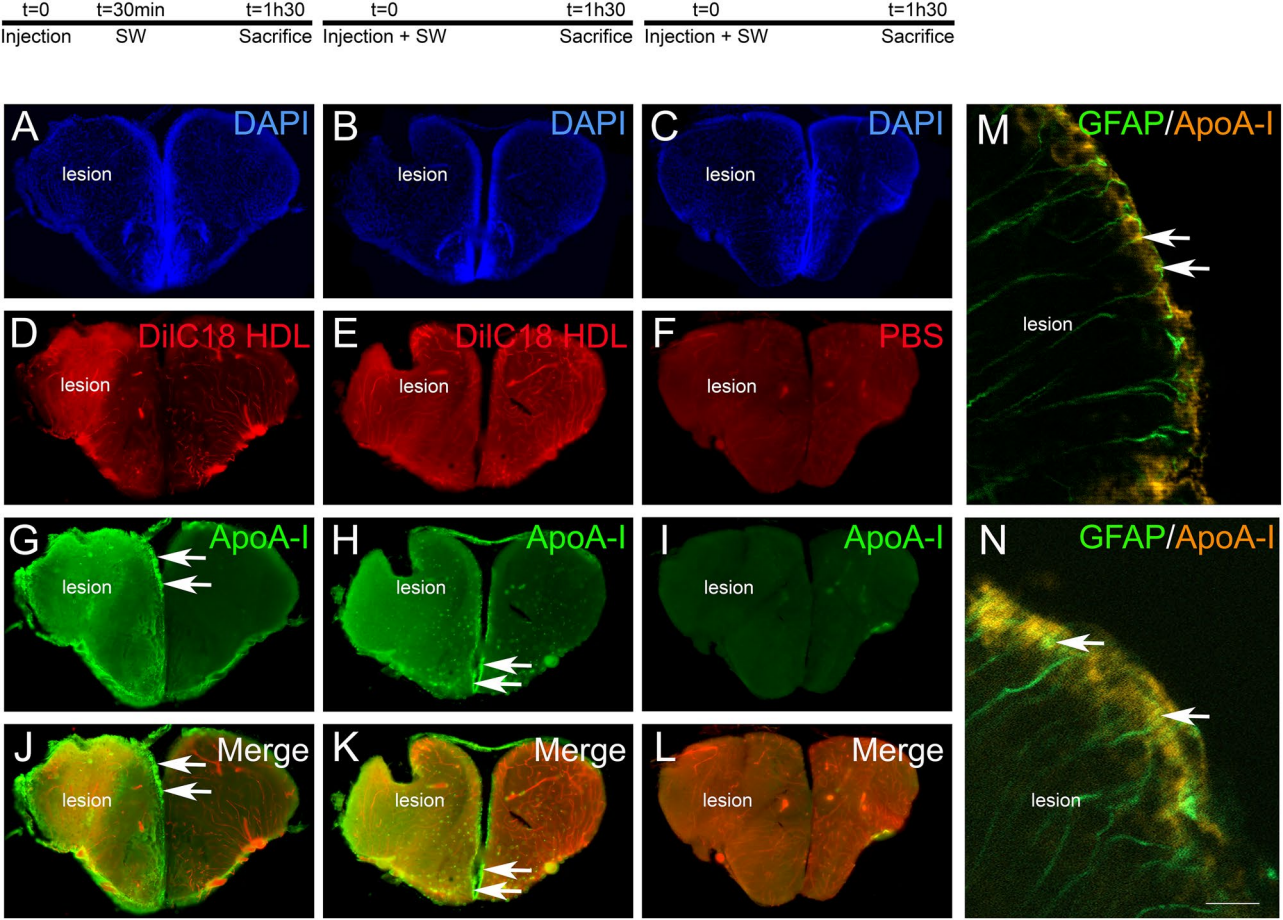
HDLs are taken up by the lesioned hemisphere after stab wound injury in zebrafish. ApoA-I immunohistochemistry (green) on injured zebrafish brain following intraperitoneal injection with 80 mg/kg of red fluorescent reconstituted HDLs (n = 8) or PBS (n = 6) as control. The design of the experiment is presented at the top of the figure. (**A**–**L**) Note that red fluorescence (HDL) and ApoA-I staining (green) are localized within the brain parenchyma of the injured hemisphere, while HDLs are clearly located in the cerebral vasculature in the contralateral (uninjured) hemisphere. The arrows indicate ApoA-I staining in ventricular cells, probably corresponding to NSCs. In PBS-injected fish (n = 3), ApoA-I immunohistochemistry did not provide any staining, demonstrating the specificity of the labeling. (**M**–**N**) ApoA-I immunohistochemistry on GFAP::GFP transgenic fish^55^ injected with HDLs immediately after the lesion. The arrows show GFAP-positive NSCs co-labeled with ApoA-I. Scale bar = 200 μm (**A**–**H**) and 20 μm (**I**, **J**).

However, after telencephalon stab wound injury, intraperitoneally injected HDLs can reach the damaged brain parenchyma as shown by DilC18 staining and ApoA-I immunostaining in the injured hemisphere compared to the uninjured one (Fig. 6). To investigate the possible differences in the biodistribution of HDLs following a preventive or therapeutic approach, two different protocols were realized. In the first one, HDL injection occurred prior to the injury, while in the second one, it occurred 30 min after the lesion. In both conditions, fluorescent HDLs diffused within the damaged tissue and ApoA-I staining was markedly increased (Fig. 6), while no staining was observed in PBS-injected fish, showing the specificity of labeling (Fig. 6I). Notably, strong ApoA-I labelling was observed in ventricular cells, resembling NSCs (Fig. 6, arrows). By performing ApoA-I IHC on GFAP::GFP fish, we demonstrated the uptake of HDLs by GFAP-positive NSCs in these conditions (Fig. 6M, N). In conclusion, in homeostatic conditions, HDLs were taken up by endothelial cells in the brain vasculature, while after brain injury, they diffused within the damaged brain hemisphere and accumulated in the ventricular layers, particularly in RGCs.

### Role of HDLs in constitutive and regenerative neurogenesis

**Figure 7:**
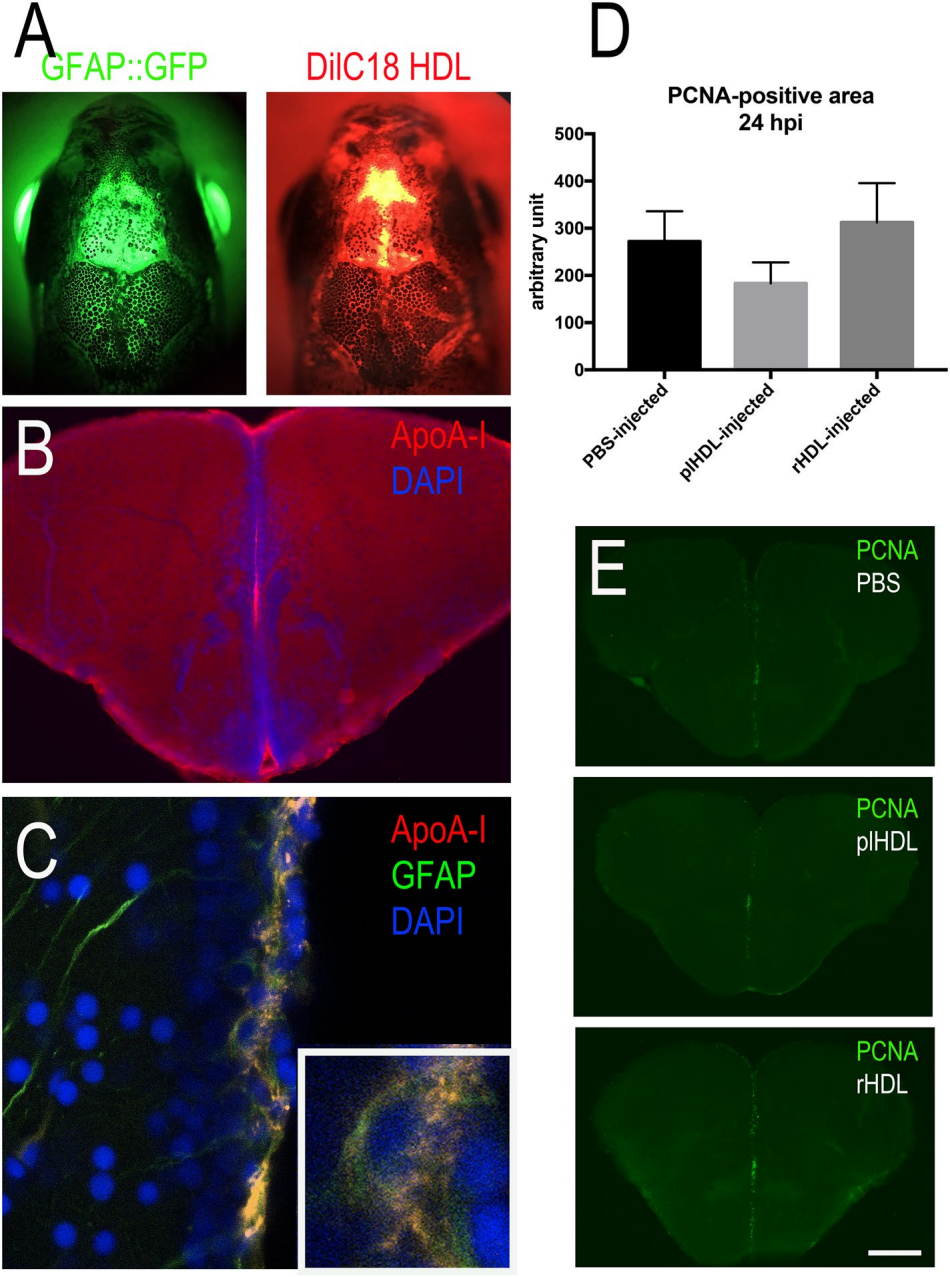
Intracerebroventricular injection of HDLs results in their uptake by neural stem cells but did not modify their proliferation. (**A**) Overview pictures of GFAP::GFP (green) zebrafish heads following injection with fluorescent HDLs (red). Note the diffusion of HDLs in the telencephalic ventricle. (**B**) ApoA-I immunohistochemistry 3 hpi showing ApoA-I detection in the ventricular cavity and the ventricular zone. (**C**) ApoA-I immunohistochemistry showing ApoA-I detection (yellow) in GFAP::GFP-positive radial glial cells (NSCs in green) with DAPI cell nuclear counterstaining (blue). (**D**) PCNA-positive area quantification following PBS, plasmatic HDL (plHDL), and reconstituted HDL (rHDL) 24 h intracerebrovascular post-injection (n = 4–6 injected fish). No significant differences were observed between the groups. (**E**) Representative pictures of PCNA immunostaining 24 h post-injection with PBS, plasmatic HDL (plHDL), reconstituted HDL (rHDL). Scale bar = 800 μm (**A**), 150 μm (**B**), 21 μm (**C**), and 200 μm (**E**).

To study the impact of HDLs on NSC proliferation during constitutive neurogenesis, intracerebroventricular injections were performed in WT and GFAP::GFP transgenic fish, in which GFP was expressed in NSCs^55^. Telencephalic proliferation was monitored 24 h post-injection as previously described^58^. The quality of the injection was assessed using fluorescent HDLs and/or by performing immunohistochemistry against ApoA-I on transgenic fish (Fig. 7A–C). As shown in freshly injected fish, fluorescent HDLs were observed in the telencephalic ventricular cavity (Fig. 7A). From 3 h to 1 day post-injection, ApoA-I immunohistochemistry was detected in the ventricular cavity and also in GFAP-positive NSCs (Fig. 7B,C), while no staining was observed in PBS-injected fish (data not shown). Quantification of the PCNA-positive area in the medial telencephalic ventricular zone of PBS-, plHDL-, and rHDL-injected fish revealed no significant difference in telencephalic brain cell proliferation (Fig. 7D,E).

**Figure 8:**
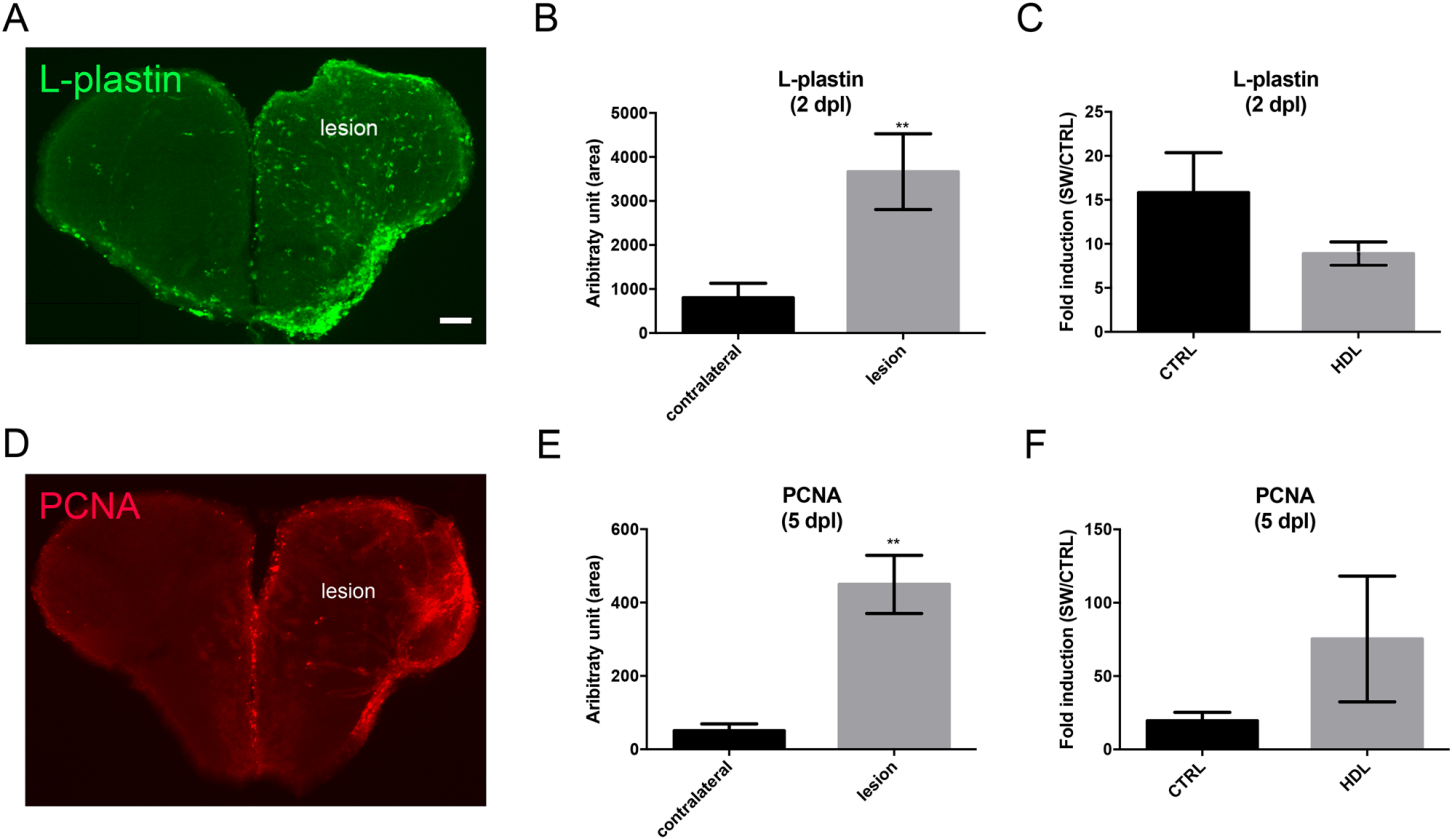
HDL injection did not impact microglia recruitment and injury-induced proliferation at the ventricular zone after telencephalic injury. Zebrafish underwent stab wound injury of the telencephalon and were injected with 80 mg/kg of plasma HDL (n = 9) or PBS as a control (n = 10) and sacrificed at 2- or 5-days post lesion (dpl). (**A**) l-Plastin immunohistochemistry (green) showing increased microglial recruitment in the injured telencephalon at 2 dpl. (**B**) Quantification of the l-Plastin-positive area in the contralateral and injured hemisphere in PBS-injected fish, demonstrating significant upregulation of microglia recruitment following brain injury at 2 dpl. (**C**) l-Plastin fold induction between lesioned and non-lesioned hemispheres in HDL and PBS-injected fish at 2 dpl. (**D**) PCNA immunohistochemistry (red) showing increased ventricular proliferation in the injured telencephalon at 5 dpl. (**E**) Quantification of the PCNA-positive area in the contralateral and injured hemisphere in PBS-injected fish, demonstrating a significant upregulation of proliferation following brain injury at 5 dpl. (**F**) PCNA fold induction between lesioned and non-lesioned hemispheres in HDL and PBS injected fish at 5 dpl. **p < 0.01. Scale bar = 100 μm.

After stab wound injury of the telencephalon, an increase in microglia proliferation and recruitment to the damaged site occurred 2 days post lesion (dpl)^47,72^. This initial step was followed by the proliferation of NSCs at 5 dpl^40,47,58^. Given the anti-inflammatory and neuroprotective roles of HDLs, their effects on brain repair mechanisms were thus studied. Intraperitoneal injections of plHDLs were performed immediately after stab wound injury. First, we checked whether stab wound injury of the telencephalon resulted in increased microglia recruitment and NSC proliferation at 2 and 5 dpl, respectively (Fig. 8A,B,D,E). As shown in Fig. 8B, a significant increase in microglia was observed at 2 dpl in the injured hemisphere compared to the control side. Similarly, proliferation in the ventricular zone was also higher in the stab wounded hemisphere compared to the uninjured one (Fig. 8E). However, injection of plHDLs directly after the stab wound did not impact microglial recruitment at 2 dpl and injury-induced proliferation at 5 dpl (Fig. 8C,F).

**Figure 9:**
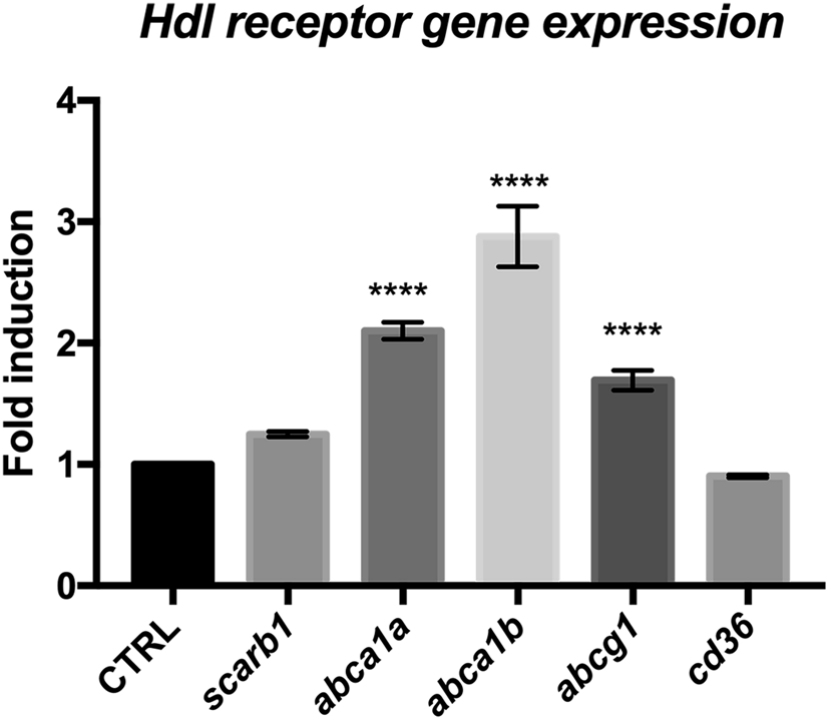
HDL receptors are upregulated at 5 days post brain injury. Reanalysis of the RNA sequencing data set from zebrafish of injured and uninjured telencephalon at 5 days post-lesions (data from^58,61^). ****p < 0.001.

Finally, the expression of *HDL receptors* was also monitored at 5 dpl, and as shown in Fig. 9, most of the HDL receptors were markedly upregulated with the exception of *scarb1,* whose expression was increased but failed to reach statistical significance, and *cd36,* for which expression remained unchanged.

### Use of HDLs as a delivery vector to target neural stem cells

**Figure 10:**
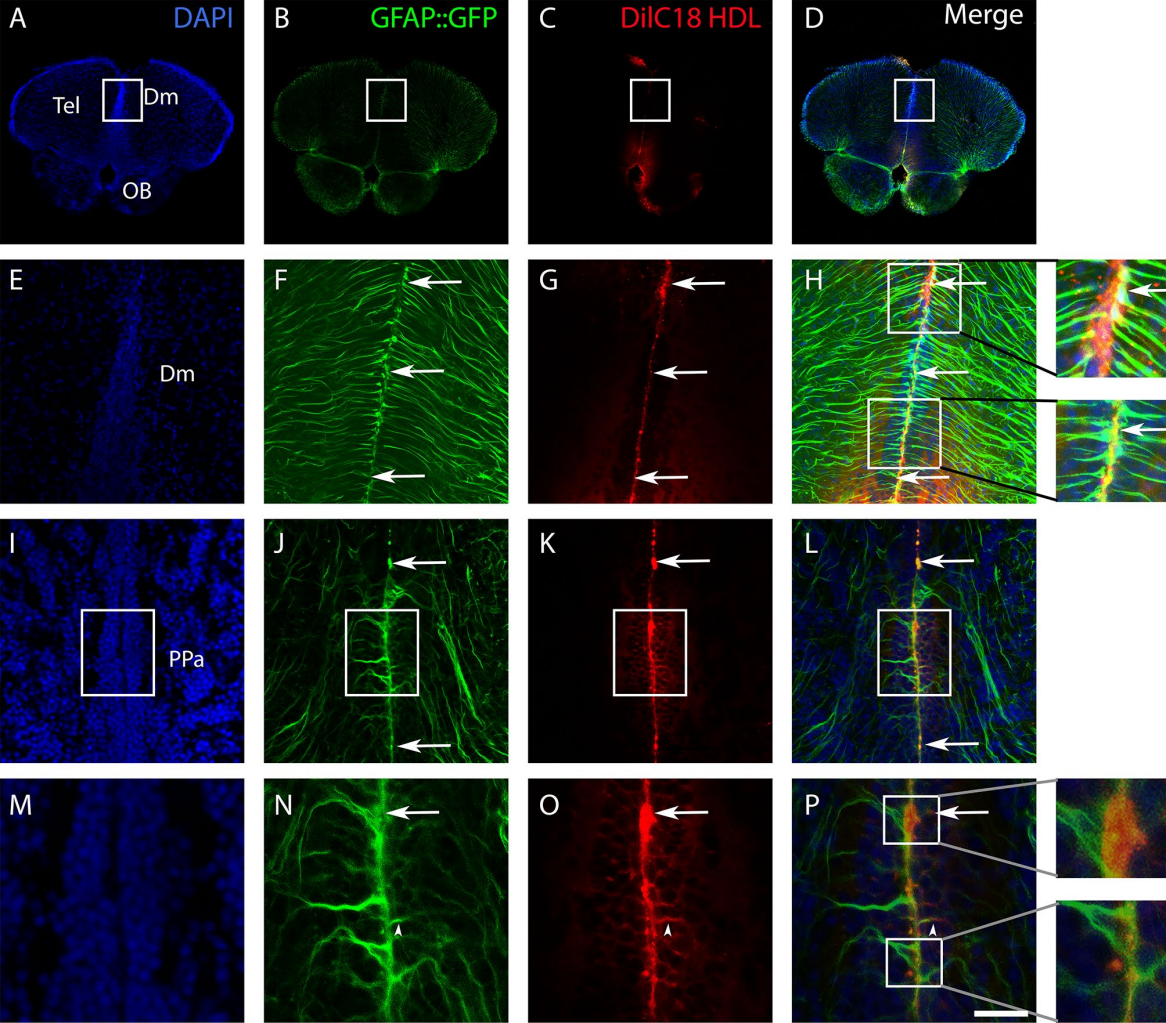
Delivery of DilC18 dye in GFAP::GFP-positive neural stem cells mediated by HDL intracerebroventricular injection. Three adult transgenic zebrafish (GFAP::GFP) were intracerebroventricularly injected with HDLs in which DilC18 dye was incorporated (fish were allowed to survive for 1 h 30 min). (**A**–**D**, **I**–**L)** Zebrafish brain sections at the junction through the olfactory bulbs (OB)/telencephalon (Tel) and through the anterior part of the preoptic area (PPa), showing DilC18 dye (red) along the ventricular layer where NSCs (green) are located. Note the uptake of the DilC18 dye (red) by NSCs (green) as shown by the yellow color in the merged pictures (see arrows). (**E**–**H**, **M**–**P)** Higher magnifications of the white boxes in (**A**–**D**, **I**–**L**), respectively. The arrows show colocalization of the dye with GFP, and arrowheads show the colocalization in neural stem cell processes. Scale bar = 700 μm (**A**, **B**), 140 μm (**E**–**H**), 85 μm (**I**–**L**), and 40 μm (**M**–**P**).

Although HDLs did not impact NSC proliferation (Fig. 7), these results highlight the fact that they could be used to target NSCs, as Apoa-I was detected by IHC in GFAP::GFP-positive cells. To ascertain the fact that HDLs could be used as a delivery vector, a fluorescent dye (DilC18) was incorporated into HDLs, and intracerebroventricular injection was performed (Fig. 10). As clearly evidenced, the dye was detected along the ventricular layer, where NSCs are located in the anterior part of the telencephalon (Fig. 10A–H), as well as in the anterior part of the preoptic area (Fig. 10I–P). The DilC18 dye was clearly observed in the GFAP::GFP-positive NSC soma (Fig. 10, see arrows) and in some processes (Fig. 10, see arrowheads).

## Discussion

In this work, we first demonstrated that HDL receptors are widely expressed in the brain of adult zebrafish. Interestingly, *scarb1* appears to be mainly expressed in both neurons and radial glial cells that are known to be *bona fide* neural stem cells. We also showed that both HDLs isolated from human plasma and reconstituted HDL particles (rHDLs made of human ApoA-I and phosphatidylcholines) are able to reach the brain vasculature in normal conditions and to diffuse within the injured hemisphere after mechanical injury of the telencephalon. They accumulate in the ventricular cell layer composed of radial glial cells and other neural progenitors. Additionally, we performed intracerebroventricular injection of HDLs to study their potential effects on NSC proliferation in ‘homeostatic’ conditions. Although intracerebroventricular injection of HDLs did not result in significant differences in telencephalic cell proliferation, it targeted NSCs and can be used as vector to modulate their proliferative activity.

### HDL receptor gene expression and links with neurogenic niches

To our knowledge, this is the first report documenting the expression of *HDL receptors* in the brain of a teleost fish. Performing RNA sequencing analyses and in situ hybridization for HDL receptors, we reported cerebral expression of *abca1a, abca1b, abcg1, cd36,* and *scarb1* in adult zebrafish. Although *abca1b* was shown to be expressed by RNA sequencing and qPCR methods, we were unable to show consistent staining by in situ hybridization experiments, probably due to the lack of sensitivity of the digoxigenin-labeled probes or unexpected splicing. We showed that *abca1a, abca1b, abcg1, cd36,* and *scarb1* were widely expressed in the brain and displayed an overlapping distribution, in particular, in most posterior regions. Their distribution was similar to that of neurons, and they were detected in the main zebrafish neurogenic niches, such as the telencephalic, diencephalic, and rhombencephalic areas. In mammals, HDL receptors are expressed in neurons and glia (microglia, astrocytes, oligodendrocytes, and their precursors), as well as endothelial cells^25^. The Allen Brain Atlas also documents the expression of HDL receptors in the mouse hippocampus^24^, a region known to maintain substantial neurogenic activity during adulthood. The nature of the cells expressing these different receptors remains to be investigated in zebrafish.

Furthermore, we demonstrated for the first time that *scarb1* is strongly expressed along the main ventricular and periventricular layers from the most anterior part of the brain to the more dorsal ones. Performing fluorescence in situ hybridization for *scarb1* in two neurogenic regions, the dorsal telencephalon and caudal hypothalamus, we proved that some *scarb1*-positive cells were Aromatase B-positive radial glial cells, corresponding to NSCs. In the same line, *scarb1* was shown to be expressed by NSC culture^26^, but to our knowledge, no functional studies have tested the impact of HDLs on neurogenesis. In our work, by performing proliferation studies following intracerebroventricular injection of rHDLs and plHDLs allowed the direct action of HDLs within the brain and in particular on radial glial cells (RGCs) acting as NSCs. We observed the uptake of HDLs by RGCs, but we did not observe any impact on telencephalic brain cell proliferation. Similarly, after stab wound injury of the telencephalon, injury-induced proliferation of NSCs was similar between fish injected with HDLs and PBS. These data suggest that human HDLs did not significantly impact zebrafish brain cell proliferation. We also considered the possibility that the human ApoA-I protein may be unable to fully activate the HDL canonical signaling pathways in zebrafish as the sequence homology between human ApoA-I and zebrafish ones (apoa1a and apoa1b) is not good (less than 30% of identity and less than 50% of similarity). Nevertheless, the expression of HDL receptors in neurogenic niches, as well as their uptake by NSCs, argue for a potential role of HDLs in brain plasticity. This idea could be supported in part by the fact that knockout mice for SR-BI display decreased hippocampal synaptic plasticity during aging linked to cognitive defects in recognition and spatial memories^73^, supporting the idea that HDLs could be important for brain plasticity, especially when considering aging. Taken together, these data support (1) the role of HDLs in brain plasticity and NSC activity and/or metabolism and (2) the possibility to use HDLs as vector to target NSCs for drug delivery to boost their neurogenic activity.

### A role for HDLs in brain repair mechanisms?

In mammals, HDLs have been shown to display anti-inflammatory and neuroprotective effects^10,19,20^. To ascertain their potential role in decreasing neuroinflammation after a stab wound injury and to promote activation of NSCs in zebrafish, we quantified microglia recruitment and telencephalic ventricular cell proliferation 2 and 5-days post-injury, respectively. These time points correspond to the peak of microglia recruitment and NSC activation following stab wound injury^40,47,58,72^. We did not observe any impact of HDLs in these processes compared to controls, in our experimental conditions. However, the injection of HDLs prior or quickly after the injury led to the diffusion of HDLs within the brain parenchyma and to their accumulation in ventricular cells resembling NSCs. This interesting result raises the question of the use of HDLs for promoting drug delivery to NSCs to favor brain repair. HDLs are complex macromolecular lipoproteins transporting different lipids and proteins, such as sphingosine-1 phosphate or paraoxonase 1, in addition to ApoA-I, that account for their endothelial protective properties^5^. Different molecules may be required in zebrafish to activate HDL-dependent protective pathways. However, not much is known concerning the structure and components of HDL particles in zebrafish.

After telencephalic brain injury in zebrafish, the RNA sequencing data demonstrated an increase in *abca1, abcg1,* and *scarb1* gene expression in the ipsilateral hemisphere compared to the control hemisphere at 5 dpl. In mouse, it was also shown that some HDL receptors were upregulated following stroke. Moreover, *abca1* gene expression was increased in reactive astrocytes from 2 to 6 days after MCAO^74^, and the *cd36* and *scarb1* genes were also increased 3 days after stroke^75,76^. The nature of the cells upregulating HDL receptors after stab wound injury remains to be determined in fish.

Taken together, these data suggest that HDLs could be important for sustaining some NSC properties following brain damage. Furthermore, HDLs could allow the vectorization of drugs to target NSCs for improving the replacement of dead neurons following brain injury. Additionally, the upregulation of HDL receptors after brain insult argues for the key role of lipoproteins and cholesterol in brain remodeling for ensuring membrane and synaptic integrity. It also raises the question of the role of HDLs as a source of cholesterol in the central nervous system.

### HDLs as a source of cholesterol for de novo steroidogenesis

The brains of fish, amphibians, and mammals are well-known to be a steroidogenic organ, able to synthesize its own steroids from cholesterol^77–81^. Radial glial cells acting as NSCs were shown to express the whole set of steroidogenic enzymes and are envisioned as true steroidogenic cells in fish^79,82,83^. Notably, the fact that such cells express *scarb1* and appear to take up HDL after stab wound injury reinforces the idea that HDLs could be a source of cholesterol for supporting NSC steroidogenesis. Such a cholesterol supply for ensuring steroidogenic functions was previously described in some other tissues in mammals^84–86^. One could argue that plHDLs were not detected in NSCs in homeostatic conditions but only in cerebral endothelial cells. However, numerous studies have shown that HDLs can cross the BBB via transcytosis in mammals^87,88^. Furthermore, a strong link between neurogenic niches and blood vessels was shown in both mammals and fish^80,89^. Furthermore, in zebrafish, it was clearly demonstrated that RGC end feet wrapped blood vessels^80^, arguing for potential exchanges and signaling between endothelial cells and NSCs, as shown recently with neuroblasts^90^. HDLs may undergo transcytosis in endothelial cells before being taken up by NSCs end feet for promoting steroidogenesis.

It is also known that the CNS increases steroidogenesis after injuries^91,92^. In a traumatic brain injury model in rats, the levels of several steroids were increased in the brain (i.e., pregnenolone, progesterone, and 5α-dihydroprogesterone)^93^. Additionally, steroids such as estrogen and progesterone have been shown to display neuroprotective effects in mammals after CNS injury^91,94–96^. However, there is no data available concerning steroidogenesis following brain injury in zebrafish. In addition, we observed an increase in HDL receptor gene expression 5 dpl in zebrafish, suggesting an increase in HDL capture after stab wound injury. Such an increase was also observed for *abca1* following brain injury (ischemia) in mammals^74^. It could be hypothesized that HDL uptake by neurogenic cells may serve for steroidogenesis in a way similar to mammals.

### HDL biodistribution in zebrafish is comparable to mammals

In mammals, it is well established that HDLs are mainly catabolized by many organs, such as the liver and kidneys^12^. Here, we showed that human plHDLs and rHDLs injected intraperitoneally were detected in both the liver and kidneys of adult zebrafish in a way similar to what we observed in mice (Suppl. Fig. 1). We also demonstrated that HDLs could reach the brain vasculature and accumulated in cerebral endothelial cells in both fish and mice (Fig. 5; Suppl. Fig. 2). Although in mammals the smaller circulating HDL particles can reach the brain parenchyma^66,67^, we were not able to detect HDL particles within the brain parenchyma in homeostatic conditions in fish. However, following brain injury, HDLs diffused into the brain parenchyma of the injured telencephalon and seemed to accumulate in the telencephalic ventricular cells known to be NSCs. These observations are particularly interesting given that after MCAO in mice, HDLs have been shown to be taken up by endothelial cells and astrocytes^19^. Moreover, the brain of adult zebrafish is devoid of astrocytes, and radial glial cells are suggested to support the functions of astrocytes participating, for instance, in the maintenance of the BBB^53,80^ and supporting a part of steroidogenesis^79,81,82,97^. At the end of mouse embryonic development, most radial glial cells disappear and are transformed into astrocytes^98^, while they persist during adulthood in zebrafish and express a set of well-characterized astrocytic markers (i.e., GFAP, vimentin, nestin). Consequently, this data supports some evolutionary conserved features in the uptake of HDLs by glial cells, being mainly internalized by astrocytes in mammals and by radial glial cells in zebrafish. They also show that HDLs may preferentially target NSCs instead of neurons.

## Conclusion

In conclusion, this work demonstrates the expression of HDL receptors in the brain of adult zebrafish, their modulation after cerebral injury, and the uptake of HDLs at the injured site. It reinforces the interest of using zebrafish for better understanding the role of lipoproteins in brain plasticity. Notably, this study also suggests the use of HDLs for the vectorization of potential neuroprotective molecules, their screening, and their impact on NSC activity. Given the upregulation of HDL receptors following brain damage in both mice and zebrafish, it raises the question of a potential beneficial effect of HDL treatment in the different phases of brain repair. Despite the well-documented neuroprotective properties of HDLs in mammals, we were not able to demonstrate, in this heterologous model and in our experimental conditions, any effect of HDLs on inflammation, microglial expression, and cell proliferation in the brain of zebrafish after a stab wound injury.

## Supplementary Information

Supplementary Information.
