# Supplementary material for: HDL biodistribution and brain receptors in zebrafish, using HDLs as vectors for targeting endothelial cells and neural progenitors
Source: Sci Rep. 2021 Mar 19;11:6439. doi: 10.1038/s41598-021-85183-9 (PMC7979862; doi:10.1038/s41598-021-85183-9)
Supplement: Supplementary file 1 — Supplementary Information. [file 41598_2021_85183_MOESM1_ESM.pdf]

HDL biodistribution and brain receptors in zebrafish, using HDLs as vectors for targeting endothelial cells and neural progenitors

Nora Cassam Sulliman[1], Batoul Ghaddar[1], Laura Gence[1], Jessica Patche[1], Sepand Rastegar[2], Olivier Meilhac[1,3], Nicolas Diotel*[1]

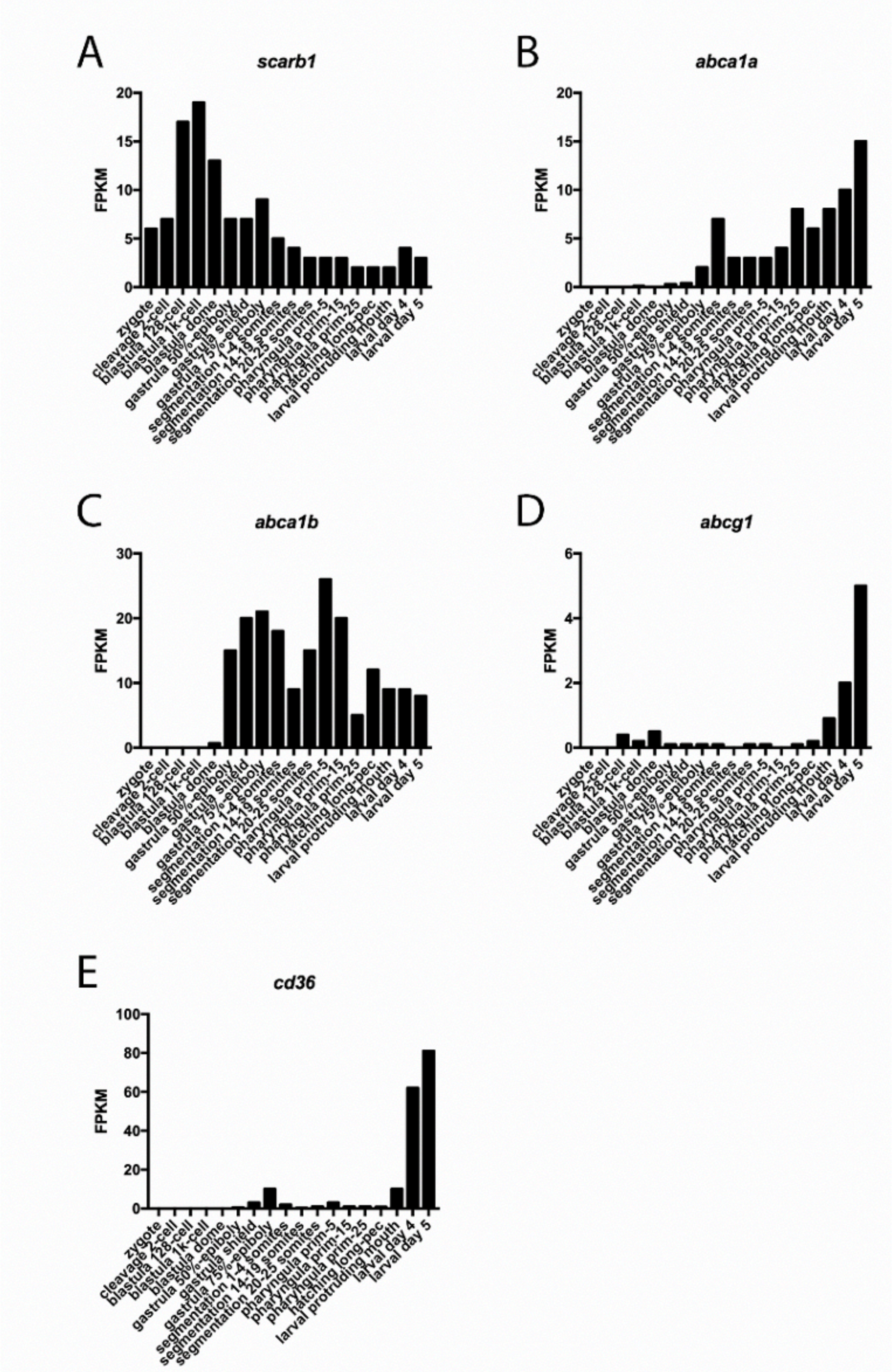

Suppl. Figure 1: **HDL** ***receptor gene expression during zebrafish development***

A-E: Transcript quantification of *scarb1, abca1a, abca1b, abcg1 and cd36* during zebrafish development between zygote stage (1-cell) to 120 hours post-fertilization (120 hpf or larval day 5). Note that these data were obtained from the reanalysis of a RNA seq data set performed by (White et al., 2017). FPKM: Fragments Per Kilobase Million.

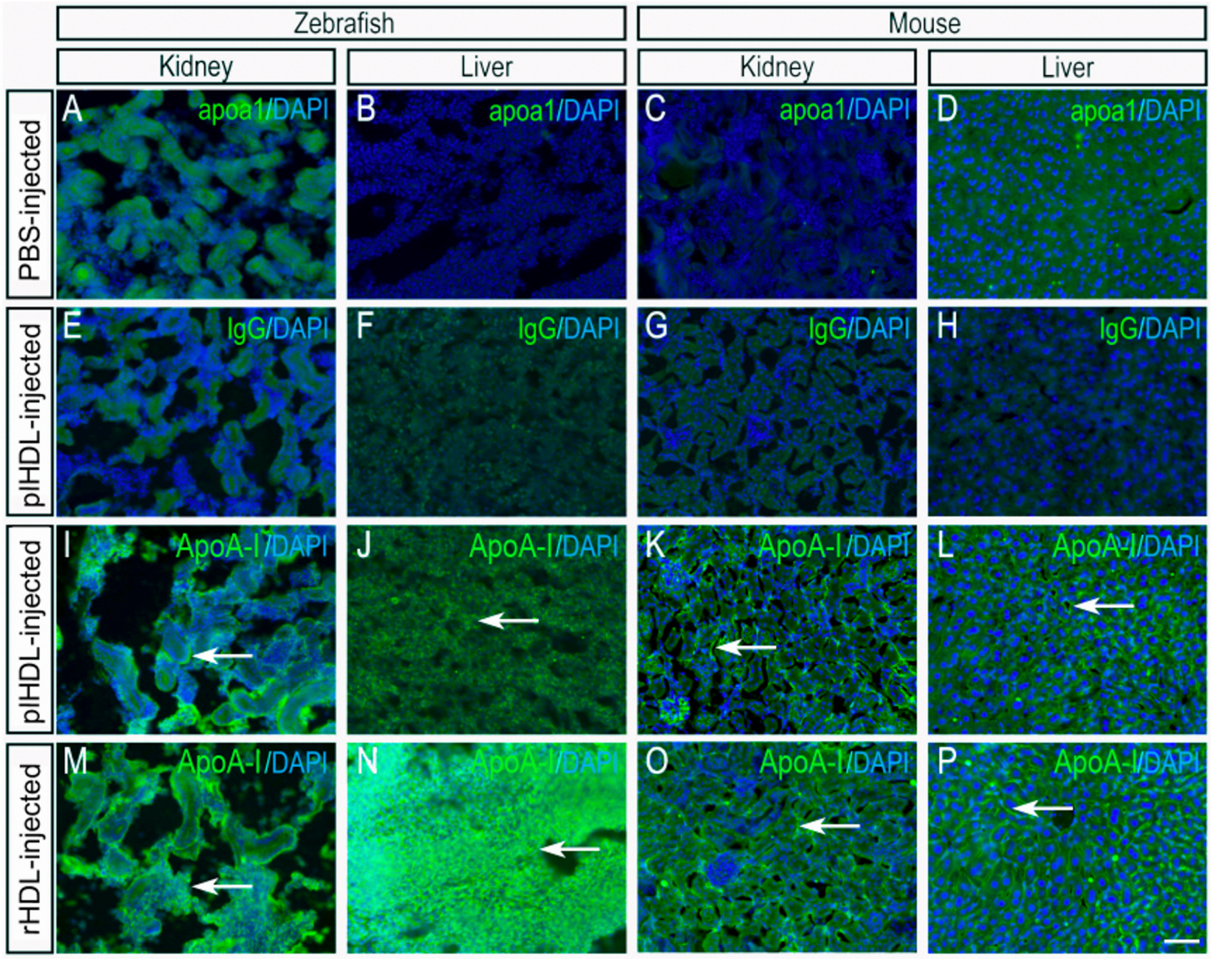

Suppl Figure 2: HDL biodistribution is similar in the liver and the kidney of mice and zebrafish. ApoA-I immunohistochemistry (green) and cell nuclear counterstaining with DAPI (blue) in liver and kidney tissue sections of zebrafish (n=4) and mouse (n=3) injected with HDL (80 mg/kg) or PBS as control and sacrificed at 1h30. Zebrafish and mice were intraperitoneally injected with PBS **(A-D)**, with plasma HDL **(E-L)**, or reconstituted HDL particles **(M-P)**. **(A-D)** No ApoA-I immunostaining was observed in PBS injected animals. **(E-H)** Immunochemistry using a control rabbit IgG on HDL injected animals results in no labeling demonstrating the specificity of the staining. **(I-P)** A positive ApoA-I signal is observed in the kidney and the liver of both zebrafish and mice injected with plasma or reconstituted HDLs. Cell nuclear counterstaining with DAPI (blue). Arrows show positive staining in green. Scale bar = 60 µm.

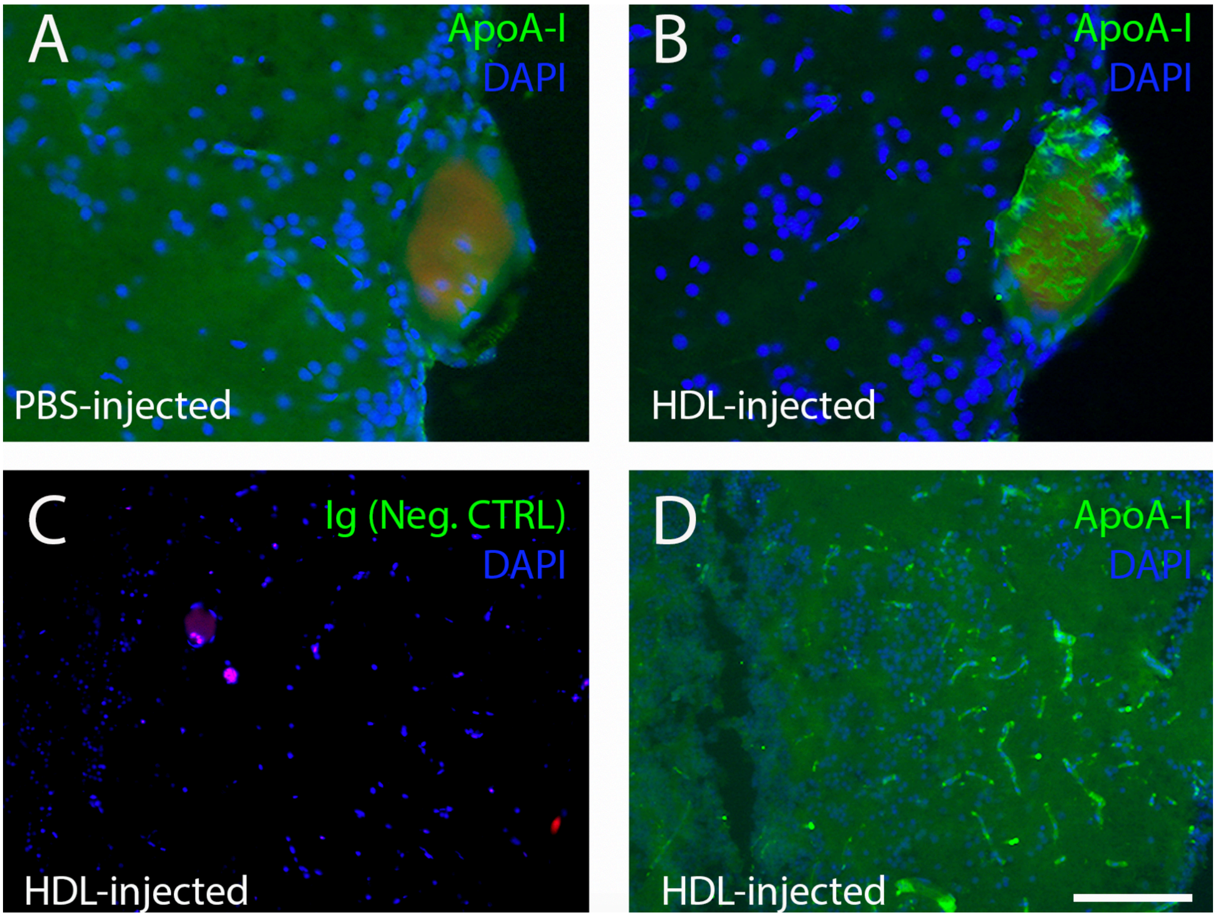

Suppl Figure 3: HDL particles reach the brain microvasculature in zebrafish

ApoA-I immunohistochemistry (green) on zebrafish brain intraperitoneally injected with HDLs (**C-D**, 80 mg/kg) or PBS (**A**) as control and sacrificed 1h30 post injection (n=3). **(A)** Absence of ApoA-I staining in PBS-injected zebrafish. **(B and D)** ApoA-I blood vessel labeling following HDL injection. **(C)** Negative control using non-immune Ig in fish injected with HDLs did not lead to any unspecific labeling. Note that all the controls demonstrate the specificity of ApoA-I labeling in the brain of adult zebrafish.

Scale bar = 70 µm (A and B) and 140 µm (C and D).

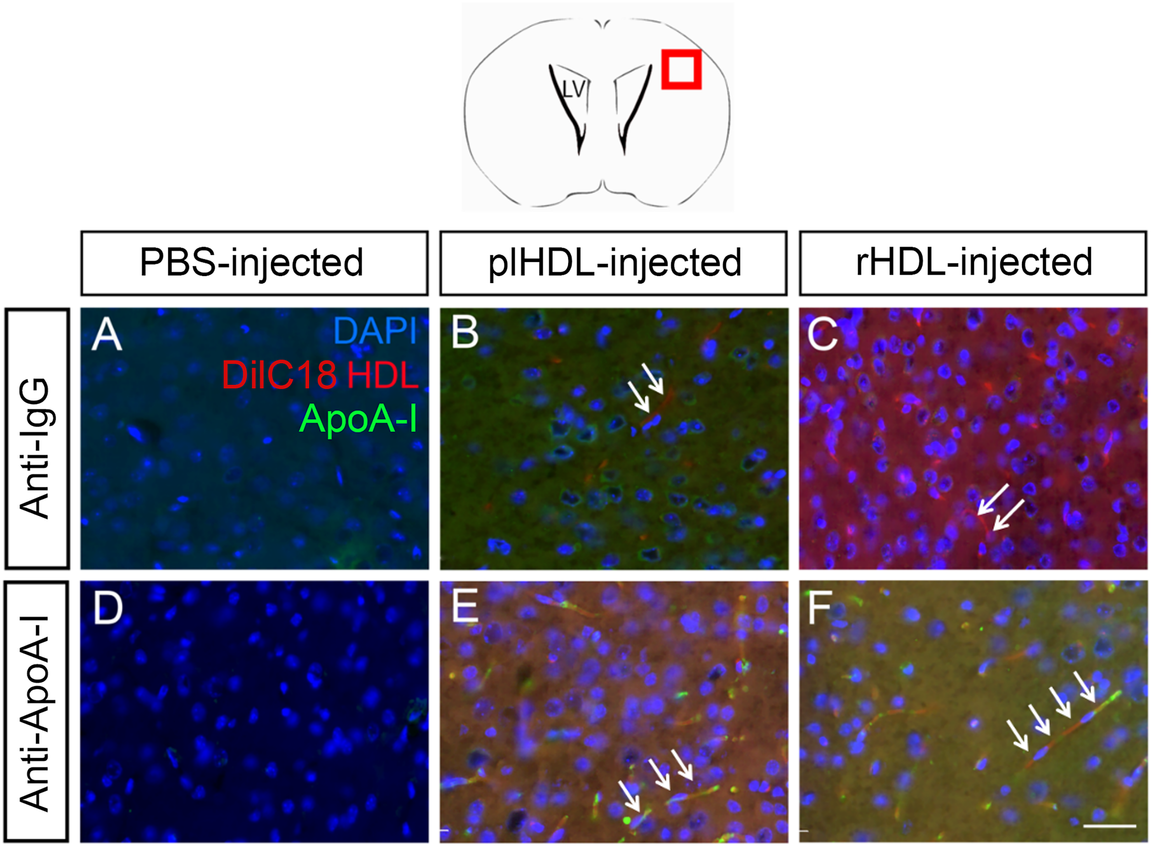

Suppl Figure 4: HDL particles reach the brain microvasculature in mice.

ApoA-I immunohistochemistry (green) on mice brain intraperitoneally injected with DilC18 HDLs (red, 80 mg/kg) or PBS as control and sacrificed 1h30 post injection (n=3). **(A-C)** Immunohistochemistry using a non-immune rabbit IgG instead (negative control) showing the absence of ApoA-I staining in green. Note that some blood vessels are labeled in red (see arrows). **(D-F)** Immunochemistry using antibody against ApoA-I (green) showing colocalization with DilC18 HDLs in blood vessels (see arrows). Scale bar= 35µm.
